# 1722. *In vivo* Activity of Imipenem/XNW4107 Human-Simulated Regimen against Serine Carbapenemase-Producing *Acinetobacter baumannii* and *Pseudomonas aeruginosa* in the Neutropenic Murine Thigh Infection Model

**DOI:** 10.1093/ofid/ofac492.1352

**Published:** 2022-12-15

**Authors:** Andrew J Fratoni, Angela Berry, Haitao Yuan, Xiao Liu, Xi Chen, Yuchuan Wu, David P Nicolau, Kamilia Abdelraouf

**Affiliations:** Hartford Hospital, Hartford, Connecticut; Hartford Hospital, Hartford, Connecticut; Evopoint Biosciences Co., Ltd, Beijing, Beijing, China; Evopoint Biosciences Co., Ltd, Beijing, Beijing, China; Evopoint Biosciences Co., Ltd, Beijing, Beijing, China; Evopoint Biosciences Co., Ltd, Beijing, Beijing, China; Hartford Hospital, Hartford, Connecticut; Hartford Hospital, Hartford, Connecticut

## Abstract

**Background:**

Imipenem (IPM)/XNW4107 is a novel β-lactam/β-lactamase inhibitor with i*n vitro* activity against serine carbapenemase-producing *Acinetobacter baumannii*, *Pseudomonas aeruginosa* and Enterobacterales. Herein, we evaluated the *in vivo* activity of an IPM/XNW4107 human-simulated regimen (HSR) against clinical OXA-23- and OXA-24-producing *A. baumannii* as well as KPC- and GES-producing *P. aeruginosa* using a neutropenic murine thigh infection model.

**Methods:**

Seven *A. baumannii* and 4 *P. aeruginosa* isolates were included. IPM and IPM/XNW4107 MICs (XNW4107 fixed at 8 mg/L) were tested in triplicate by broth microdilution. One thigh of neutropenic ICR mice (6 mice per group) was inoculated with ∼10^7^ CFU/mL bacterial suspensions. HSR that mimicked the clinical exposures of IPM 500 mg q6h alone or in combination with XNW4107 250 mg q6h each as 1 h infusion were developed in the murine model. In efficacy studies, two hours after inoculation, placebo, IPM 500 mg q6h 1 h infusion HSR, or IPM/XNW4107 500/250 mg q6h 1 h infusion HSR were administered subcutaneously. Efficacy was measured as the change in log_10_CFU/thigh at 24 h compared with 0 h controls.

**Results:**

Isolates were IPM resistant (MICs 16 - > 64 mg/L). IPM/XNW4107 *A. baumannii* and *P. aeruginosa* MIC ranges were 1-16 and 1- 8 mg/L, respectively. Across all examined isolates, 0 h mean ± SD bacterial burden was 5.86 ± 0.32 log_10_ CFU/thigh. The 24 h increase in bacterial burden was 2.68 ± 0.91 log_10_ CFU/thigh in the sham controls. IPM HSR monotherapy groups showed mean increase in bacterial burden of 2.34 ± 0.95 log_10_ CFU/thigh. Bacterial kill with IPM/XNW4107 500/250 mg q6h 1 h infusion HSR ranged from -0.46 ± 1.69 to -3.77 ± 0.15 and -2.33 ± 0.25 to -3.76 ± 0.57 among *A. baumannii* and *P. aeruginosa* isolates, respectively. IPM/XNW4107 500/250 mg q6h 1 h infusion HSR produced > 1-log kill against 6/7 examined *A. baumannii* with the exception of *A. baumannii* 160 (IPM/XNW4107 MIC 16 mg/L) and 4/4 *P. aeruginosa* as well as > 2-log kill against 4/7 *A. baumannii* and 4/4 *P. aeruginosa*.

**Conclusion:**

IPM/XNW4107 500/250 mg q6h 1 h infusion HSR showed potent *in vivo* activity against serine carbapenemase-producing *A. baumannii* and *P. aeruginosa*. These data support the consideration of IPM/XNW4107 for the treatment of serious infections due to these organisms in clinical trials.

**Disclosures:**

**Haitao Yuan, PhD**, Evopoint Biosciences Co., Ltd: Stocks/Bonds **Xiao Liu, PhD**, Evopoint Biosciences Co., Ltd: Stocks/Bonds **Xi Chen, PhD**, Evopoint Biosciences Co., Ltd: Stocks/Bonds **Yuchuan Wu, PhD**, Evopoint Biosciences Co., Ltd: Stocks/Bonds **David P. Nicolau, PharmD**, Shionogi: Grant/Research Support **Kamilia Abdelraouf, PhD**, Evopoint Biosciences Co., Ltd: Grant/Research Support|Venatorx Pharmaceuticals, Inc.: Grant/Research Support.

